# Foot-and-mouth disease virus VP1 target the MAVS to inhibit type-I interferon signaling and VP1 E83K mutation results in virus attenuation

**DOI:** 10.1371/journal.ppat.1009057

**Published:** 2020-11-24

**Authors:** Pathum Ekanayaka, Seo-Yong Lee, Thilina U. B. Herath, Jae-Hoon Kim, Tae-Hwan Kim, Hyuncheol Lee, Kiramage Chathuranga, W. A. Gayan Chathuranga, Jong-Hyeon Park, Jong-Soo Lee

**Affiliations:** 1 College of Veterinary Medicine, Chungnam National University, Daejeon, Republic of Korea; 2 Animal and Plant Quarantine Agency, Gyeongsangbuk-do, Republic of Korea; 3 FVC, Gyeongsangbuk-do, Republic of Korea; 4 Laboratory Animal Resource Center, Korea Research Institute of Bioscience and Biotechnology, University of Science and Technology (UST), Daejeon, Republic of Korea; 5 Infectious Disease Research Center, Korea Research Institute of Bioscience and Biotechnology, Daejeon, Republic of Korea; 6 California Institute for Quantitative Biosciences, University of California, Berkeley, California, United States of America; Duke University Medical Center, UNITED STATES

## Abstract

VP1, a pivotal capsid protein encoded by the foot-and-mouth disease virus (FMDV), plays an important role in receptor-mediated attachment and humoral immune responses. Previous studies show that amino acid changes in the VP1 protein of cell culture-adapted strains of FMDV alter the properties of the virus. In addition, FMDV VP1 modulates host IFN signal transduction. Here, we examined the ability of cell culture-adapted FMDV VP1(83K) and wild-type FMDV VP1(83E) to evade host immunity by blocking mitochondrial antiviral signaling protein (MAVS)/TNF Receptor Associated Factor 3 (TRAF3) mediated cellular innate responses. Wild-type FMDV VP1(83E) interacted specifically with C-terminal TRAF3-binding site within MAVS and this interaction inhibited binding of TRAF3 to MAVS, thereby suppressing interferon-mediated responses. This was not observed for cell culture-adapted FMDV VP1(83K). Finally, chimeric FMDV harboring VP1(83K) showed very low pathogenicity in pigs. Collectively, these data highlight a critical role of VP1 with respect to suppression of type-I IFN pathway and attenuation of FMDV by the E83K mutation in VP1.

## Author summary

Foot-and-Mouth disease (FMD), a highly contagious viral disease of cloven-hoofed animals, causes huge economic losses. To generate a FMD vaccine, cell culture-adapted strains of FMDV that show improved growth properties and allow repeated passage are needed. Generally, adaptation of field-isolated FMDV is accompanied by changes in viral properties, including amino acid mutations. A VP1 E83K mutation in cell culture-adapted FMDV was identified previously; here, we examined the impact of VP1 E83K on virus pathogenicity and type-I IFN pathway. Cell culture-adapted FMDV O1 Manisa, and highly virulent strain of O/Andong/SKR/2010, acquired the E83K mutation in the VP1 protein, which attenuated the virus via disposing VP1 mediate negative regulation ability of host cellular IFN responses. The data suggest a rational approach to viral propagation in cell culture and virus attenuation, which could be utilized for future development of FMDV vaccines.

## Introduction

Foot-and-mouth disease (FMD) virus is the causative agent of a highly infectious disease with a huge economic impact; the virus infects cloven-hoofed mammals, including cattle, swine, and more than 70 species of wild animal [1,2]. FMDV is a member of the genus *Aphthovirus* within the family *Picornaviridae* [3]; there are seven classified FMDV serotypes (O, A, Asia1, C, SAT1, SAT2, and SAT3) based on the antigenicity of the structural proteins, and a range of strains within each serotype [1,4]. However, the clinical symptoms caused by the different serotypes are almost identical [5,6]. FMDV has a positive sense RNA genome of 8.5 kb in length, which contains a single open reading frame encoding four structural proteins (VP4, VP2, VP3, and VP1) and ten non-structural proteins (L, 2A, 2B, 2C, 3A, 3B1–3, 3C, and 3D); these proteins are expressed with the help of virus-encoded proteases [4,7]. The FMDV capsid comprises 60 copies of the structural proteins [8]; among them, VP1 is critical for cell attachment and host immunity as it contains epitopes critical for induction of humoral immune responses [9–15].

VP1 facilitates viral attachment to host epithelial cells by binding to adhesion receptors. *In vivo*, FMDV attaches to host cells via an arginine-glycine-aspartic acid (RGD) sequence in the G-H loop of VP1 [13,16–18]; this sequence binds to any of four integrins (αυβ1, αυβ3, αυβ6, and αυβ8) that act as receptors [19–25] on the surface of susceptible cells. In addition, FMDV can also enter cells through non-integrin-mediated pathways; for example, cell culture-adapted FMD viruses attach to host cells via heparan sulfate (HS), which is a cell surface glycosaminoglycan [26,27]. Following attachment, the virus is internalized and its genome is released into the cytosol [28,29]. It is thought that cellular virus receptors exert important selective pressure, thereby enabling viruses to adapt via mutation [30,31]. Serial passage of viruses in cultured cells leads to evolution of cell culture-adapted mutated viruses [32]; some of these mutations can either attenuate or increase virulence [27,33].

Upon infection, viruses induce production of cytokines, including type-I interferons (IFNs), and subsequent activation of downstream molecules that trigger host antiviral innate immune responses [34–37]. The invading viral RNA is recognized by cellular cytosolic pattern recognition receptors such as retinoic acid-inducible gene I (RIG-I), melanoma differentiation associated protein 5 (MDA5), and toll-like receptors (TLRs) [38,39]. RIG-I in particular is a key cytosolic sensor that recognizes 5’-triphosphate-containing double stranded RNA from diverse viruses or short double stranded RNA molecules [40–45]; activation of RIG-I induces structural modifications that permit CARD-CARD interactions with the downstream adapter molecule, mitochondrial antiviral signaling protein (MAVS; also known as IPS-1, VISA, and Cardif) [42,43,46,47]. This then activates type-I interferon responses via downstream signaling molecules TBK1/IKKε, IRF3, IRF7, and NF-κB (activated via IKK) to elicit inflammatory responses [42,43,46,47].

FMDV is very sensitive to type-I interferon [48–53]. Thus, FMDV has acquired a number of strategies to evade host type-I interferon responses [29,54]. FMDV VP1 can block the type-I interferon pathway [55,56], although the exact mechanism underlying FMDV VP1-induced suppression of type-I interferon production is unclear.

Cell culture adaptation of wild-type viruses involves functional changes in viral proteins [57]; consequently, cell culture-adapted viruses lose the ability to suppress IFN. For example, cell culture-adapted measles virus proteins P and V show reduced ability to inhibit IFN-β signaling [57]. Furthermore, previous studies show that cell culture-adapted FMDV strain harbors amino acid substitution E83K, which is not present in the VP1 region of the field strain [58,59]. Even though the E83K mutation in VP1 is of considerable interest, it has been studied only in the context of particle assembly [60,61] and cell surface receptor adhesion [58,59].

Based on previous evidence, we demonstrate herein that FMDV VP1 mediates a novel negative regulation mechanism, and report the impact of the VP1 E83K substitution in the context of cellular type-I IFN responses and attenuation of FMDV in a pig model.

## Results

### Wild-type FMDV VP1(83E) negatively regulates antiviral immune responses

Previous studies show that wild-type FMDV VP1 antagonizes the type-I IFN pathway [55,56]. Furthermore, FMDV VP1 acquires the 83K point mutation upon cell culture [58,59].

Based on current knowledge, we compared the effect of point mutated FMDV VP1(83K) and wild-type (83E) of O1/Manisa/Turkey/69 (O1 Manisa) and O/Andong/SKR/2010 (Andong) strains on antiviral immune response pathways in porcine kidney (PK-15) cells. The O1 Manisa is a mostly used FMDV vaccine strain that having low-pathogenicity features. The Andong strain is a highly pathogenic and virulent FMDV strain. Also, genomic RNA of O1 Manisa strain is used as the virus backbone which we used for the generation of FMDV chimeric virus. Based on those reasons, we used FMDV VP1 of both O1 Manisa and Andong strains to compare its virus replication and type-I IFN inhibition phenotypes in the *in-vitro* model.

**Fig 1:**
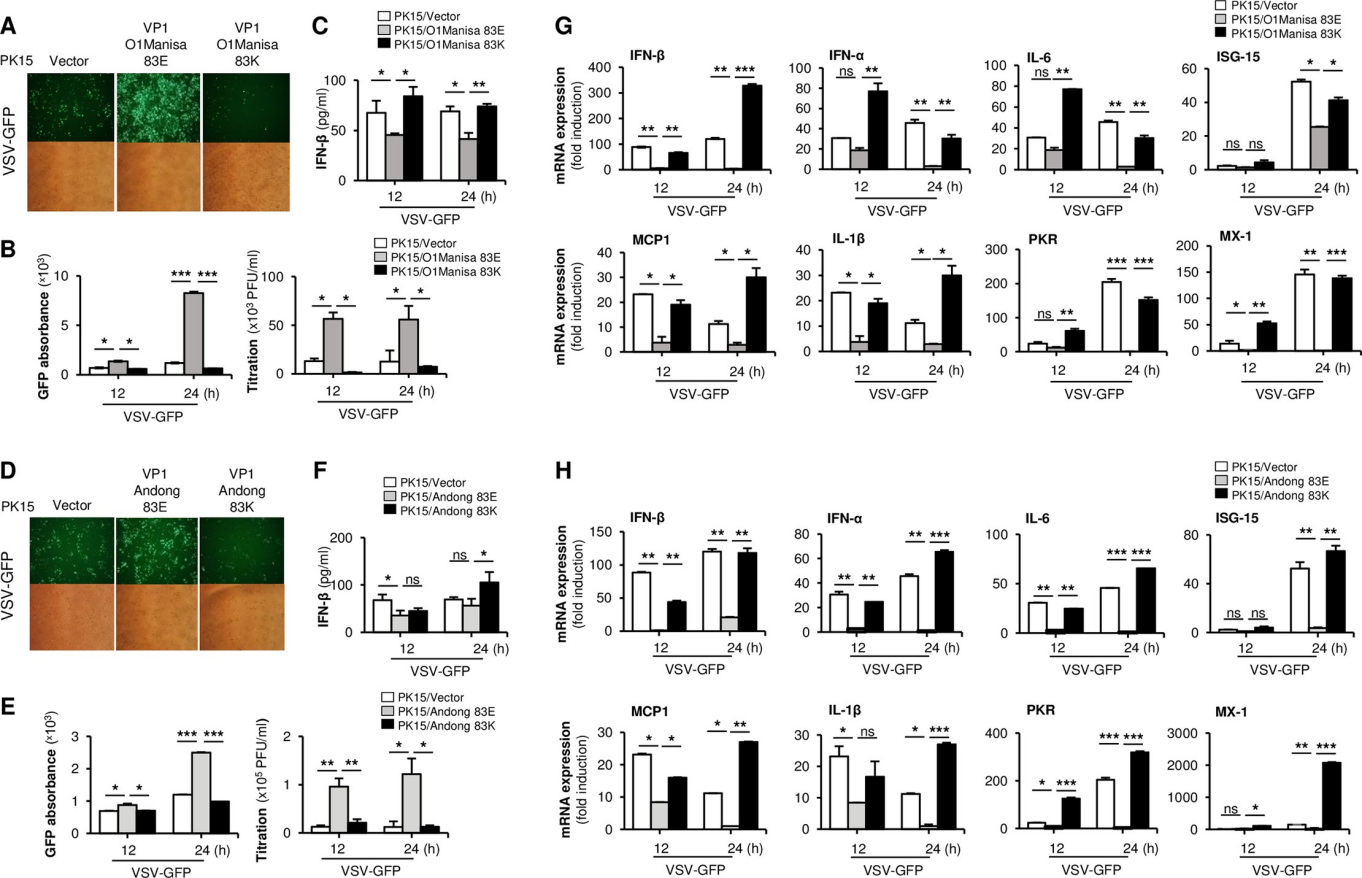
Wild-type FMDV VP1 negatively regulate type-I IFN pathway. PK15 cells were transiently transfected with wild-type VP1 and VP1 83K of FMDV O1 Manisa strain along with control vector, and VSV-GFP (1MOI) were infected. (A) GFP expression, (B) GFP absorbance and virus titer, and (C) IFN-β secretion was measured at indicated time points. The same experiment was conducted for the wild-type VP1 and VP1 83K of O/Andong/SKR/2010 (Andong) FMDV strain and similarly, (D) GFP expression, (E) GFP absorbance and virus titer, and (F) IFN-β secretion was measured. (G-H) Wild-type VP1 and VP1 83K of FMDV O1 Manisa and Andong strains along with control vector were transiently transfected to the PK15 cells, and VSV-GFP (1MOI) was infected. At 12 and 24hpi, cells were harvested and analyzed by quantitative real-time PCR analysis for IFN-β, IFN-α, IL-6, ISG-15, MCP1, IL-1β, PKR, and MX-1 genes. Data are representative of three independent experiments, each with similar results. Error bars indicate the mean ± SD of two biological replicates. Student’s t test; *p < 0.05; **p < 0.01; ***p < 0.001; ns, not significant.

For that, the cells were transiently transfected with a control plasmid, or with plasmids containing wild-type (83E) or cell culture-adapted (83K) FMDV VP1 and infected with VSV-GFP. As expected, VSV-GFP replication was higher, and IFN-β production was lower, for wild-type FMDV VP1 than for the control (Fig 1A–1F). In addition, wild-type VP1 inhibited expression of mRNA encoding IFN-β, IFN-α, and other antiviral-related genes (Fig 1G and 1H). However, cell culture acquired FMDV VP1(83K) transfection did not affect on virus replication, IFN-β production, or expression of antiviral genes upon VSV-GFP infection (Fig 1). This phenomenon was observed for both the O1 Manisa and Andong strains. The similar results of IFN-β production were observed in wild-type (83E) and cell culture-adapted (83K) FMDV VP1 transfected PK15 cells upon stimulation with poly(I:C) or 5’ppp-dsRNA (S1 Fig). These results suggest that wild-type FMDV VP1(83E) is a negative regulator of type-I IFN signaling, and cell-culture-acquired point mutation in VP1(83K) results in lose of such antagonistic ability.

### Wild-type FMDV VP1(83E) targets MAVS to suppress the type-I IFN pathway

**Fig 2:**
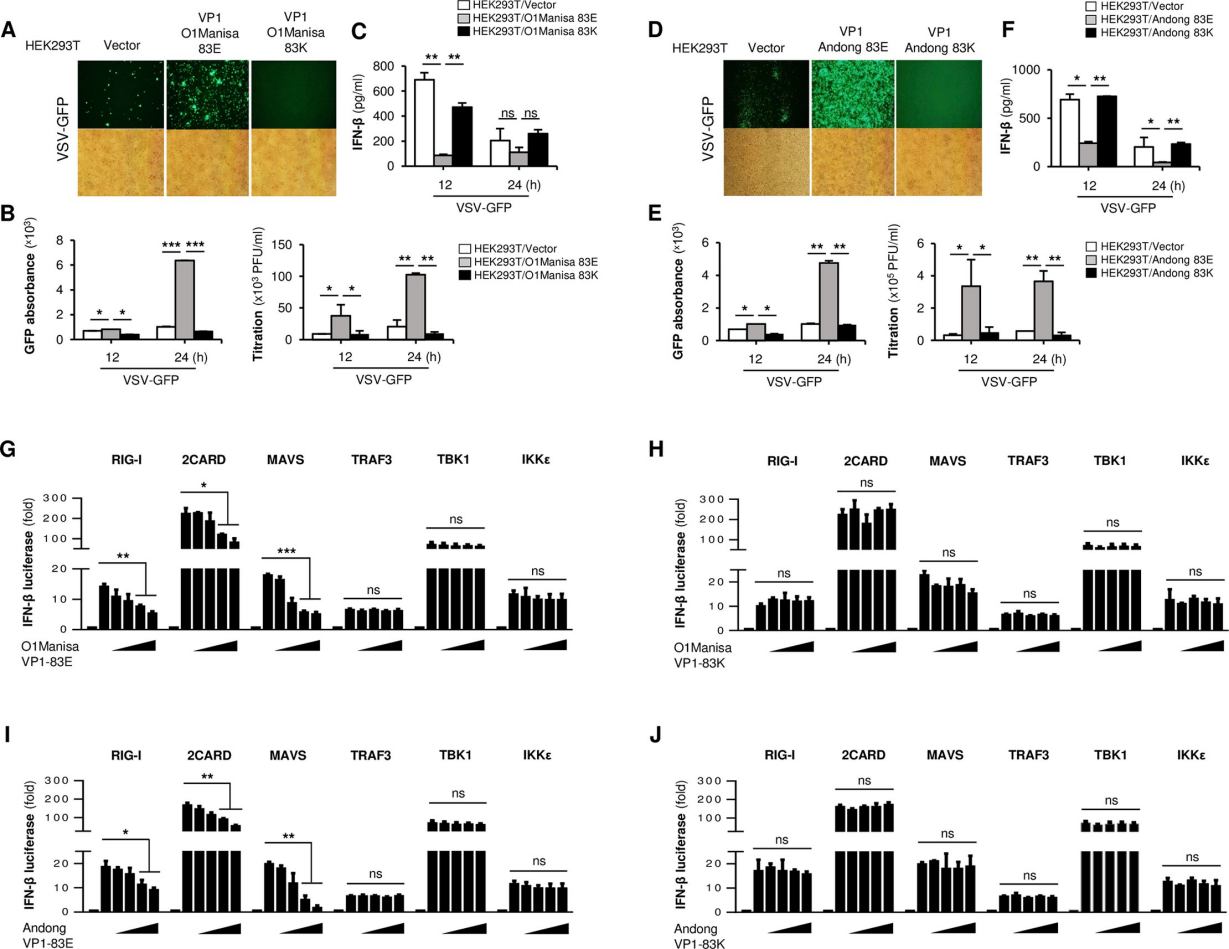
Wild-type FMDV VP1 targets MAVS to inhibits IFN-β promoter activity. HEK293T cells were transiently transfected with wild-type VP1 and VP1 83K of FMDV O1 Manisa strain along with control vector, and VSV-GFP (1MOI) were infected. (A) GFP expression, (B) GFP absorbance and virus titer, and (C) IFN-β secretion was measured at indicated time points. The same experiment was conducted for the wild-type VP1 and VP1 83K of O/Andong/SKR/2010 (Andong) FMDV strain and similarly, (D) GFP expression, (E) GFP absorbance and virus titer, and (F) IFN-β secretion was measured. (G-J) HEK293T cells were transfected with the firefly luciferase reporter plasmid encoding the IFN-β promoter, plus TK-Renilla plasmid and an increasing dose of flag-tagged (G and I) wild-type VP1 and (H and J) VP1 83K plasmids of O1 Manisa and Andong strains, plus expression plasmids for RIG-I, 2CARD, MAVS, TRAF3, TBK1 and IKK-ε, for 24h. Results are expressed relative to those of Renilla luciferase alone (internal control). Data are representative of at least two independent experiments, each with similar results. All the values are expressed as mean ± SD of two biological replicates. Student’s t test; *p < 0.05; **p < 0.01; ***p < 0.001; ns, not significant.

To further confirm the effects of FMDV VP1 on type-I IFN signaling responses, we transiently transfected HEK293T cells with a control plasmid, or with plasmids containing wild-type (83E) or cell culture-adapted (83K) FMDV VP1, followed by infection with VSV-GFP. The phenotype of HEK293T cells was similar to that of PK15 cells in that cells transfected with wild-type FMDV VP1 showed higher VSV-GFP replication and lower IFN-β production than the control. As in PK15 cells, cell-culture-acquired FMDV VP1(83K) had no influence on neither virus replication or IFN-β production; this was the same for the O1 Manisa and Andong strains (Fig 2A–2F).

Upon virus infection, host sensor molecules activate type-I IFN signaling cascades [36,37,39]. The above results, and those of previous studies, show that wild-type FMDV VP1 negatively regulates this signaling pathway [55,56]. Hence, to identify the potential target of FMDV VP1 in the type-I IFN cascade, we performed a luciferase promoter assay in HEK293T cells by co-expressing either wild-type VP1(83E) or cell culture-adapted FMDV VP1(83K) along with several IFN-related genes. We found that wild-type FMDV VP1 of the O1 Manisa and Andong strains strongly inhibited RIG-I, 2CARD, and MAVS-mediated IFN-β promoter activity in a dose-dependent manner (Fig 2G–2I). However, there was no detectable change in TRAF3, TBK1 or IKKε-mediated promoter activity with increasing expression of FMDV VP1 (Fig 2G–2I). Since TBK1 and IKKε locate in the downstream of TRAF3 and the nondetectable change in TRAF3-mediate promoter activity with the presence of VP1 suggest that wild-type FMDV VP1 targets the molecule immediately upstream of TRAF3 in type-I IFN pathway. The MAVS is the molecule which locates immediately upstream of TRAF3 and because of that, we postulate wild-type FMDV VP1 targets the MAVS signaling complex to suppress type-I IFN response. However, in agreement with the virus infection phenotype experiments above, cell culture-acquired FMDV VP1(83K) did not affect IFN-β promoter activity (Fig 2H–2J). In addition, the results in S2 Fig illustrates that FMDV VP1 doesn’t inhibit IFN receptor signaling by IFN-β treatment. These results further confirm the difference in the type-I IFN regulatory functions of wild-type (83E) and cell culture-adapted (83K) FMDV VP1, and suggest specific physical and functional interactions between FMDV VP1 and MAVS.

### Wild-type FMDV VP1(83E) targets the TRAF3 binding site of MAVS to interfere with the MAVS-TRAF3 interaction

**Fig 3:**
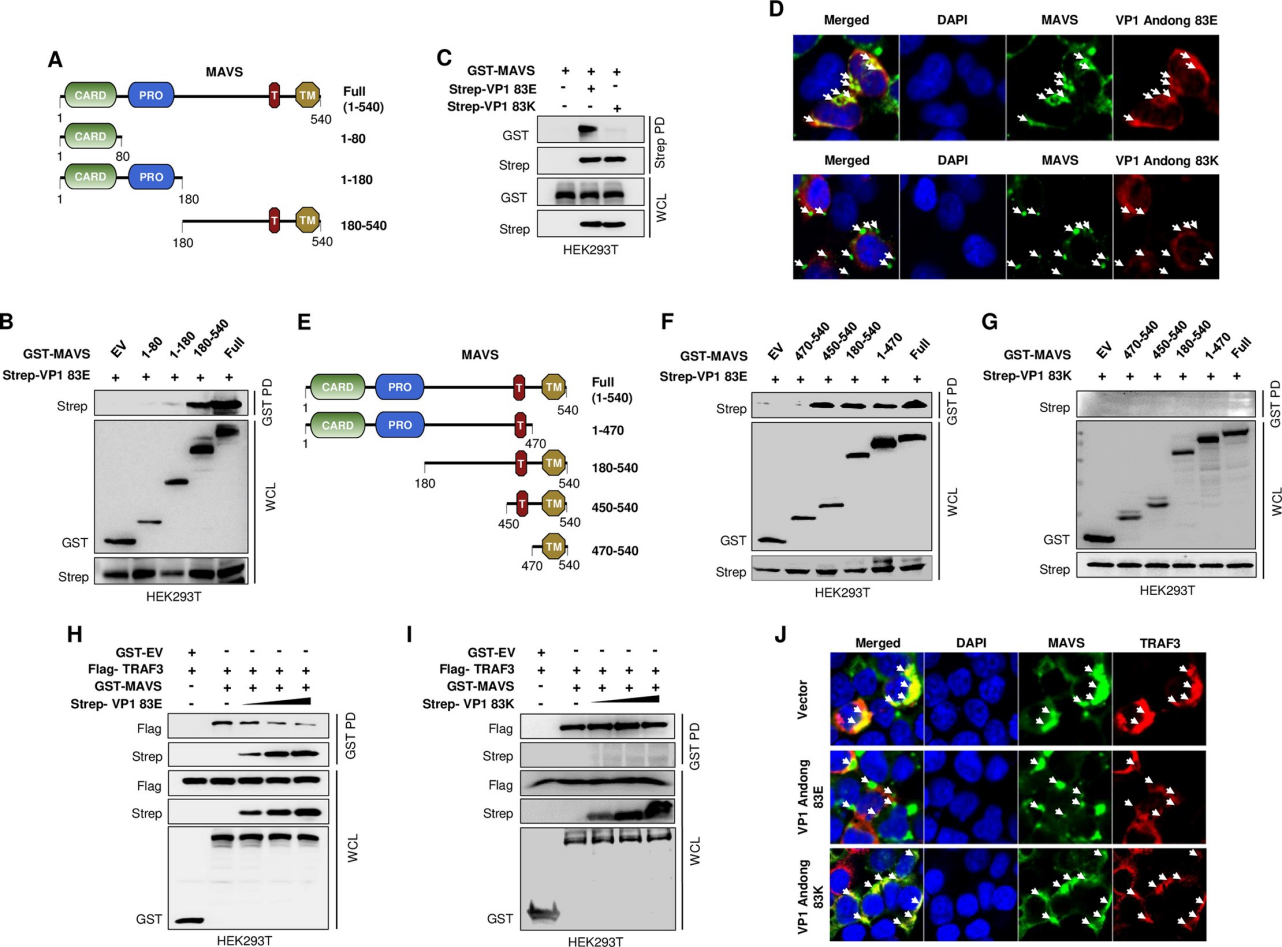
Only wild-type FMDV VP1 compete with TRAF3 to bind with MAVS. (A) Full length and deletion mutants of MAVS was subjected to (B) immunoprecipitation with wild-type FMDV VP1 (83E) of O/Andong/SKR/2010 (Andong) strain. Lysates of HEK293T cells transfected with control vector (GST) or GST-MAVS constructs comprising amino acid 1–80, 1–180, 180–540, and full form of MAVS with Strep-tagged wild-type VP1 were subjected to GST-PD (pulldown), followed by immunoblotting with an anti-Strep antibody. Whole-cell lysate (WCL) was immunoblotted with anti-Strep and anti-GST antibodies. (C) HEK293T cells were cotransfected with the control vector (Strep), GST-MAVS, and Strep-tagged wild-type (83E) and mutated (83K) VP1 plasmids of FMDV Andong strain. Cell lysates were subjected to Strep pulldown (PD), followed by immunoblotting with an anti-GST antibody. Whole-cell lysate (WCL) was immunoblotted with anti-Strep and anti-GST antibodies. (D) HEK293T cells were transfected with Flag-tagged wild-type VP1 (83E) or VP1 83K plasmids of FMDV Andong strain together with V5-tagged MAVS plasmid, followed by confocal microscopy assay with anti-Flag (red) and anti-V5 (green) antibodies. Nuclei were stained with DAPI (blue). (E) several deletion mutant constructs of GST-MAVS comprising amino acid 470–540, 450–540, 180–540, 1–470 and full-length MAVS or control vector (GST) were cotransfected to HEK293T cells together with Strep tagged (F) wild-type VP1 and (G) VP1 83K of FMDV Andong strain. Cell lysates were subjected to GST-PD and immunoblotted with anti-Strep antibody following immunoblotting of the WCL with both anti-Strep and anti-GST antibodies. (H and I) The FMDV VP1 mediate blocking of MAVS-TRAF3 association was assessed with competition assay. Simply, HEK293T cells were cotransfected with control vector (GST), Flag-TRAF3, GST-MAVS, and increasing doses of Strep-tagged (H) wild-type VP1 and (I) VP1 83K of FMDV Andong strain. The cell lysates were subjected to GST-PD and subsequent immunoblotting with anti-Flag and anti-Strep antibodies. Further, WCL was immunoblotted with anti-Flag, anti-Strep, and anti-GST antibodies. (J) HEK293T cells were transfected with Strep-tagged wild-type VP1 (83E), VP1 83K plasmids of FMDV Andong strain, or control plasmid (Strep) together with Flag-tagged TRAF3 and V5-tagged MAVS plasmid, followed by confocal microscopy assay with anti-Flag (red) and anti-V5 (green) antibodies. Nuclei were stained with DAPI (blue). All the data are representative of two independent experiments, each with similar results.

To confirm the luciferase promoter activity results, we conducted immunoprecipitation assays to examine the ability of wild-type FMDV VP1 to associate with MAVS or several MAVS deletion mutants (Fig 3A). The co-immunoprecipitation results showed a clear association between MAVS and wild-type VP1; the N-terminal 1–80 and 1–180 amino acid domains of MAVS lost the ability to interact with VP1, whereas the C-terminal amino acids (180–540) of MAVS and full-length MAVS bound strongly to VP1 (Fig 3B). In addition, the immunoprecipitation results of wild-type FMDV VP1 with RIG-I, MDA5, MAVS, TRAF3, and TBK1 molecules in the type-I IFN pathway showed the selectivity of VP1 and MAVS interaction (S3 Fig).

Further, to assess the relative importance of the glutamic acid (E) residue at position 83 within FMDV VP1 with respect to MAVS regulation, we compared the ability of wild-type (83E) and cell culture-adapted (83K) FMDV VP1 to associate with MAVS. For that, we performed immunoprecipitation assays after transfecting HEK293T cells separately with a MAVS-GST plasmid together with strep-tagged wild-type (83E) or cell culture-adapted (83K) FMDV VP1 plasmids. Interestingly, the co-immunoprecipitation results showed a clear association between MAVS and wild-type VP1 only (Fig 3C). We also used confocal microscopy to confirm the colocalization of wild-type (83E) and cell culture-adapted (83K) VP1 with MAVS (Fig 3D). This observation suggests that the glutamic acid (E) at position 83 in FMDV VP1 is important for MAVS binding.

Next, to characterize the specific MAVS motif that binds to FMDV VP1, we constructed a series of GST-tagged MAVS deletion mutants (Fig 3E) and transfected them into HEK293T cells along with a strep-tagged expression plasmid containing wild-type (83E) or cell culture-adapted (83K) FMDV VP1. We then assessed the VP1-MAVS interaction by co-immunoprecipitation. The results indicated that wild-type FMDV VP1 failed to bind the N-terminal truncated form (aa 470–540) of MAVS, but bound strongly to the full length and other truncated forms (aa 1–470, 180–540, and 450–540) of MAVS (Fig 3F). However, as expected, cell culture-adapted FMDV VP1 (83K) did not show any interaction with the full length or truncated forms of MAVS (Fig 3G). Indeed, this suggests that wild-type FMDV VP1(83E) binds predominantly to amino acids 450–470 of MAVS, which reside near the transmembrane domain. Interestingly, previous studies demonstrate that MAVS functional TRAF3-binding sites locate in its 455–460 amino acid region [62,63] that allow MAVS to recruit TRAF3 and assemble the MAVS signaling complex, resulting in activation of the type-I IFN pathway [62–64]. Thus, our results suggest that wild-type FMDV VP1 specifically targets the TRAF3 binding site (aa 455–460) of MAVS to block TRAF3 recruitment to MAVS. MAVS-mediated regulation of type-I IFN induction is achieved by direct and specific interaction with TRAF3 [65]. Thus, we performed a competition assay by transfecting HEK293T cells with different amounts of strep-tagged wild-type (83E) or cell culture-adapted (83K) FMDV VP1 plasmid together with MAVS-GST and TRAF3-Flag plasmids to investigate whether FMDV VP1 interferes with the assembly of the MAVS-TRAF3 signalosome. In the presence of increasing amounts of FMDV VP1, MAVS was immunoprecipitated by an anti-GST antibody, and TRAF3 and VP1 were detected using anti-Flag and anti-Strep antibodies, respectively. We found it interesting that competitive co-immunoprecipitation assays demonstrated that only wild-type FMDV VP1 disrupted the MAVS-TRAF3 interaction in a dose-dependent manner (Fig 3H); as expected, there was no detectable disruption of the MAVS-TRAF3 interaction by cell culture-adapted FMDV VP1 (Fig 3I). Additionaly we further confirmed the function of wild-type (83E) and cell culture-adapted (83K) VP1 on the disruption of MAVS-TRAF3 interaction by using confocal microscopy (Fig 3J and S4 Fig).

These results suggest that wild-type FMDV VP1(83E) mediates a novel mechanism of type-I IFN pathway antagonism by interrupting TRAF3 recruitment to the MAVS; cell culture-adapted FMDV VP1(83K) harboring a point mutation lacks such antagonistic ability.

### Construction and characterization of a chimeric virus

**Fig 4:**
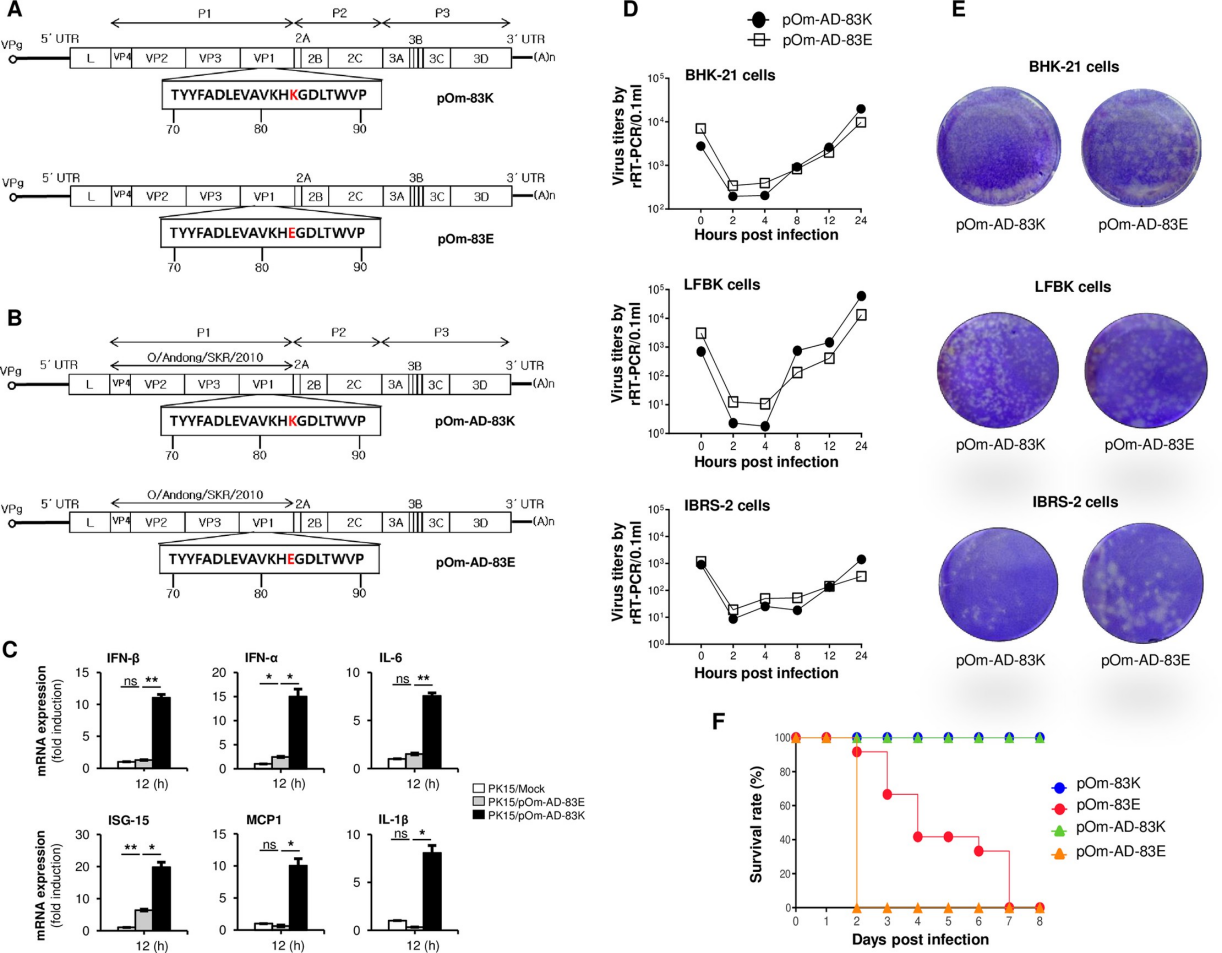
Construction and characterization of the chimeric virus. (A and B) Schematic depiction of the chimeric viruses. The chimeric clones pOm-83E and pOm-AD-83K have replaced the 83rd amino acid with 83E or 83K through point mutation, respectively. (C) PK15 cells were infected with pOm-AD-83K and pOm-AD-83E (0.05MOI), together with mock infection. At 12hpi, cells were harvested and analyzed by quantitative real-time PCR analysis for IFN-β, IFN-α, IL-6, ISG-15, MCP1, and IL-1β genes. (D) The pOm-AD-83K and pOm-AD-83E chimeric virus were infected to BHK-21, LFBK, and IBRS-2 cells. At the indicated time points virus titer was determined by RNA extraction and quantitative real-time PCR analysis. (E) BHK-21, LFBK, and IBRS-2 cells were infected with each virus at 10^5.0^~10^1.0^ TCID_50_/0.1mL with 10-fold dilutions and stained with crystal violet at 72h after virus infection. The images show the wells inoculated with each virus at 10^3.0^ TCID_50_/0.1mL. (F) For the comparison of pathogenesis in suckling mice (n = 10), the survival rate was determined after challenge with recombinant FMDVs intraperitoneally. In C, data are representative of two independent experiments, each with similar results and all the values are expressed as mean ± SD of two biological replicates. Student’s t test; *p < 0.05; **p < 0.01; ***p < 0.001; ns, not significant.

Consistent with previous studies, we found that FMDV serotype O possess a glutamic acid (E) residue at position 83 of the VP1 protein (S1 Table). Upon cell culture adaptation, FMDV serotypes O and SAT2 acquire the 83K point mutation in the VP1 protein [58,59]. However, the effect of the cell culture-acquired FMDV VP1 83K point mutation on virus pathogenicity is unknown. Therefore, we extended our study to examine the effect of the VP1 83K point mutation on FMDV pathogenicity by constructing a chimeric virus. The FMD virus O1 Manisa, a widely used vaccine strain [66], was the recombinant backbone for this study. First, the cell culture-adapted O1 Manisa strain (pOm-83K) generated by serial passage of an O1 Manisa infectious clone in BHK-21 cells to acquired the 83K point mutation in VP1. The pOm-83E was produced from the use of mutagenesis by changing the Lysine (K) to Glutamic acid (E) at the 83rd amino acid of the VP1 of pOm-83K (Fig 4A). Fig 4B shows a schematic diagram illustrating the chimeric virus genome produced by replacing the P1 region of O1 Manisa virus with that of wild-type O/Andong/SKR/2010 highly virulent strain, denoted as pOm-AD-83E. The generation of pOm-AD-83K was performed by using mutagenesis for changing E to K in the pOm-AD-83E.

To check the presence of VP1 83E and 83K related IFN production phenotypes (similar to Figs 1–3) in chimeric viruses, pOm-AD-83E and pOm-AD-83K were infected to PK15 cells by following quantitative real-time PCR analysis for detecting IFN-β and other type-I IFN pathway related gene expression. The results show the same pattern similar to Figs 1–3, that only pOm-AD-83E inhibits the antiviral gene expression including IFN-β, and pOm-AD-83K is not (Fig 4C). Additionally, to characterize the recovered viruses, we examined cytopathic effects in BHK-21, LFBK, and IBRS-2 cells up to several passages. The results indicate that the chimeric virus harboring VP1 83K showed strong cytopathic effects in cultured cells (S2 Table). Furthermore, to evaluate genetic stability, recovered viruses were passaged in BHK-21, LFBK, and IBRS-2 cells. Next, the viral genome sequences were analyzed to confirm that the 83K mutation in recovered viruses remained unchanged throughout the passages, additionally, VP1 83E eventually returned to VP1 83K through passage (S3 Table).

Moreover, to compare the growth kinetics and plaque phenotypes of the two chimeric viruses (pOm-AD-83K and pOm-AD-83E), we constructed virus growth curves and performed a plaque assay in different cultured cells. Based on the results, both chimeric viruses harboring the 83K point mutation and wild-type VP1, respectively, showed equivalent replication kinetics when tested in three cell lines (Fig 4D). On the other hand, in the plaque assay, all three cell lines had the larger plaques in wild-type VP1 containing pOm-AD-83E chimeric virus (Fig 4E). Additionally, we evaluated the growth of the chimeric viruses in BHK-21, LFBK, and IBRS-2 cell lines (up to five passages) by RT-PCR. The results showed that FMDV harboring the 83K point mutation in the VP1 region grew equally well in all cell lines at all passages. However, pOm-83E grew only in BHK-21 cells, and grew poorly in LFBK and IBRS-2 cells (S5 Fig).

*In vivo* evaluation of virulence was performed first in suckling mice. Mice were infected with recombinant viruses and survival rates were monitored. Interestingly, consistent with the *in vitro* virus replication results, all mice infected with the cell culture-adapted recombinant virus harboring the 83K point mutation in VP1 survived up until 8 days post-infection, whereas all mice infected with the pOm-AD-83E chimeric virus or pOm-83E recombinant virus died at 2 and 7 days post-infection, respectively (Fig 4F). This illustrates that the 83K point mutation within VP1 of FMDV attenuates pathogenicity.

### Cell-cultured FMDV harboring the VP1 83K point mutation shows attenuated virulence in pigs

**Fig 5:**
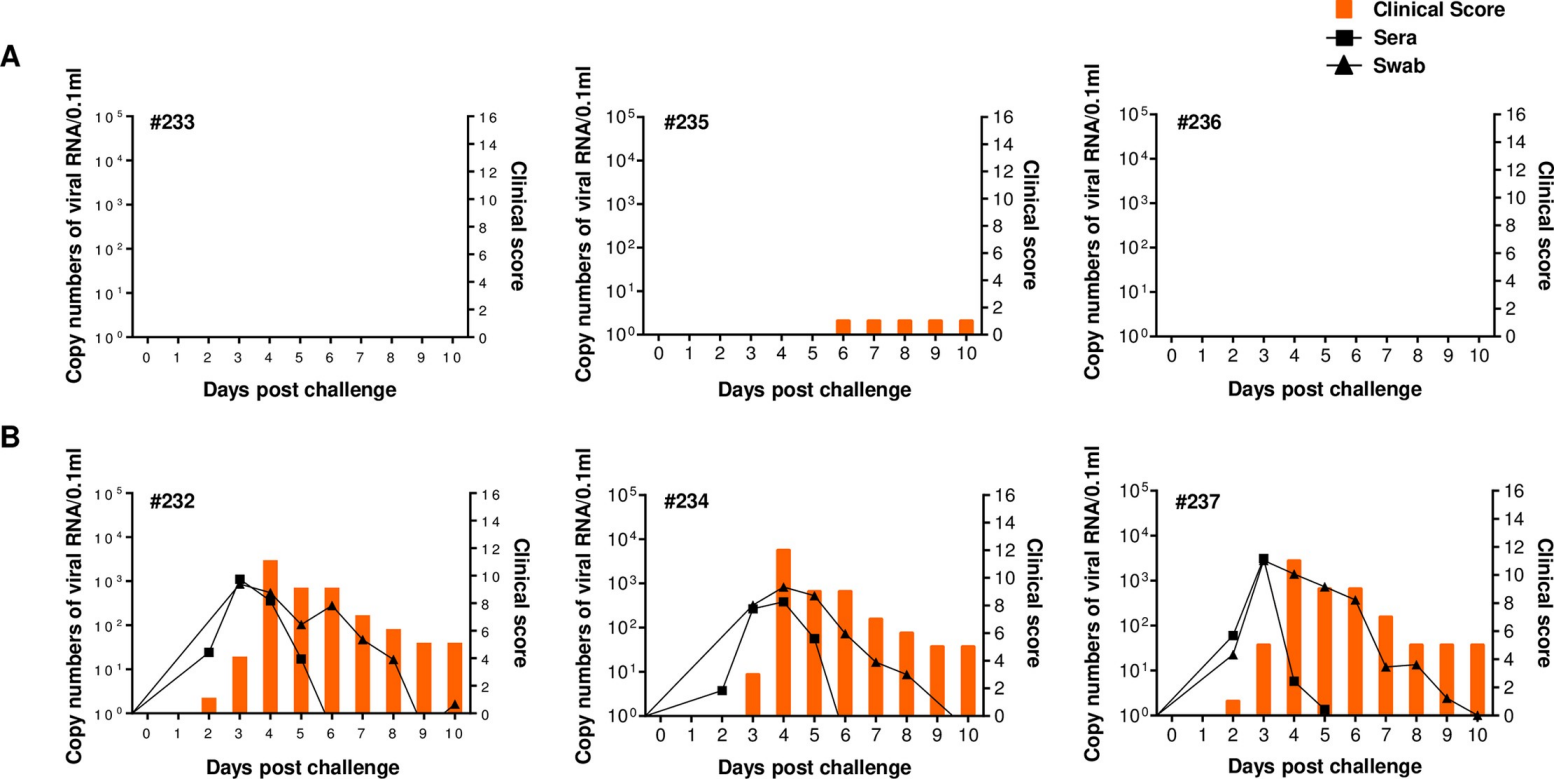
Pathogenesis of the chimeric viruses in pigs. The challenge experiment was carried out with six 90-day-old Yucatan pigs. (A) Three pigs were inoculated with pOm-AD-83K (#233, #235, #236). (B) Another three pigs were inoculated with pOm-AD-83E (#232, #234, #237). Each virus was inoculated on the footpad at a concentration of 10^5.0^ TCID_50_/0.1mL. The left Y-axis of the graph shows the amount of virus in sera and swab as log_10_ values and the right Y-axis shows the clinical index as the maximum value of 16 points.

Finally, to evaluate the effects of the 83K point mutation on FMDV pathogenicity in a natural host, pigs were challenged directly with pOm-AD-83K or pOm-AD-83E. Following challenge, several disease parameters were analyzed: clinical score, viremia, and neutralizing antibody titers. The results showed that animals (animals #233, #235, and #236) infected with pOm-AD-83K did not develop severe clinical signs of FMD. In addition, there was no virus release from sera and oral cavities (Fig 5A). By contrast, animals (animals #232, #234, and #237) infected with the pOm-AD-83E chimeric virus showed clinical signs typical of FMD, starting on Day 2 post-infection; in addition, high levels of virus were detected in serum and the oral cavity (Fig 5B). With little difference between the two chimeric viruses, neutralizing antibody titers of pOm-AD-83E and pOm-AD-83K groups increased at 5 and 6 days post-infection, respectively (Table 1).

**Table 1:** Summaries of virus neutralizing antibody level in pigs after adaptation or mutation virus challenge.

Challenge Virus	Pig ID	Serum neutralizing antibody titers against O/SKR/2010 (log10)											Virus detection by real-time RT-PCR for 10 days after challenge		Clinical score^§^
		0^†^	1	2	3	4	5	6	7	8	9	10	Sera^‡^	Swab^‡^	
**pOm-AD-83K**	233	<1.2	<1.2	<1.2	<1.2	<1.2	<1.2	1.2	2	2	2.1	2.1	-	-	0
	235	<1.2	<1.2	<1.2	<1.2	<1.2	<1.2	1.2	1.8	2.1	2.3	2.3	-	-	1
	236	<1.2	<1.2	<1.2	<1.2	<1.2	<1.2	1.3	1.8	1.7	2.1	2	-	-	0
**pOm-AD-83E**	232	<1.2	<1.2	<1.2	<1.2	<1.2	2.11	2.1	2.3	2.3	2.6	2.6	+	+	11
	234	<1.2	<1.2	<1.2	<1.2	<1.2	1.51	2.1	2.3	2.4	2.6	2.6	+	+	12
	237	<1.2	<1.2	<1.2	<1.2	1.36	1.95	2.1	2.1	2.1	2.1	2.1	+	+	11

† Day post challenge (DPC).

‡ (+) represented positive reaction of virus in sera and swab.

§ Clinical score represented sum of clinical score during experimental periods.

Collectively, these results demonstrate that the cell culture-acquired 83K point mutation in VP1 attenuates the pathogenicity of the FMD chimeric virus; these data support earlier results showing that the VP1 83K mutation fails to antagonize the type-I IFN pathway *in vitro*.

## Discussion

Type-I IFN responses are the first line of defense against virus infection [37,67]; FMDV is highly sensitive to IFNs [48–53]. Thus, FMDV has evolved the ability to antagonize type-1 IFN and evade the immune response, thereby allowing successful infection and replication in host cells [54].

The FMDV protease (mainly L^pro^ and 3C^pro^) plays a crucial role in immune evasion by targeting IFN-related signal transduction pathways and are known to shut-off the host translation system [68,69]. FMDV L^pro^ is translocated to the nucleus where it cleaves the p65 subunit of NF-ĸB [70,71], thereby suppressing IRF3/7 protein expression [72,73]; importantly, it functions as a viral deubiquitinase that deubiquitinates RIG-I, TBK1, TRAF3, and TRAF6 [74] for negative regulation of type-I IFNs. In addition to L^pro^, FMDV 3C^pro^ cleaves NEMO [75], and disrupt the nuclear translocation of STAT1 via degradation of the KPNA1 nuclear translocation signal receptor [76]; it also degrades the RIG-1, MDA5 [75], and LGP2 [77] cellular viral RNA detection receptors, all of which belong to the type-I IFN pathway. Furthermore, FMDV 3C^pro^ degrades PKR to facilitate virus replication [78], as well as suppressing autophagy and NF-ĸB antiviral responses through ATG5-ATG12 protein degradation [79]. In addition to FMDV proteases, the 3A protein is known to involve in RIG-I, MDA5 and MAVS gene transcription inhibition [80], and DDX56 mediate IRF3 phosphorylation inhibition [81]. A recent report shows that FMDV 3A inhibits expression of RIG-I and MDA5 by upregulating LRRC25-mediated G3BP1 degradation [82]. The non-structural protein FMDV 2B interacts with RIG-I and LGP2 to impair antiviral signal transduction [77,83]; however, the underlying mechanism is unknown. The FMDV structural protein VP3 inhibits MAVS protein expression by disrupting its mRNA, thereby contributing to type-I IFN pathway evasion [84,85]. Furthermore, previous studies report that FMDV structural protein VP1 suppresses the type-I IFN pathway through incorporation with sorcin [55,56].

FMDV capsid protein VP1 plays a key role in virus attachment to host cells [13,16–18]. During cell culture adaptation, mutations in residues located within the VP1 protein occur frequently and can influence the virulence of the virus [86]. Indeed, guinea pig-adapted FMDV harbors an L147P substitution in VP1, resulting in altered receptor recognition, lack of growth in different established cell lines and modify its antigenicity while not affecting its ability to cause acute and transmissible disease in pigs [87]. In addition, passage of FMDV strain Asia1/HN/CHA/06 four times in suckling mice leads to acquisition of a S154D mutation in the G–H loop of the VP1 protein, which increases viral replication and pathogenicity [88]. Similarly, virus passage in cultured cells alters pathogenicity through acquisition of mutations [32]. Previous studies report that serotype O and SAT2 of FMDV gain a E83K (Glu-Lys) point mutation in VP1 upon cell culture adaptation [58,59]. The amino acid E, originally encoded at position 83, is negatively charged, whereas cell culture-acquired K in the same position is positively charged [58]. In a cell culture system, the positively charged residue at position 83 of VP1 increases the affinity of FMDV for heparin sulfate (HS), the cell surface receptor for virus entry [58,59]. One study suggests that a positively charged residue (K) in VP1 creates a charged patch on the virus capsid surface, which facilitates clearance of viruses from the animal’s circulation system, thereby disrupting binding to the specific cell types required to produce vesicular disease [58]. Consequently, non-integrin-mediated interactions between cell surface receptors and the VP1 protein of cell culture-adapted FMDV results in virus attenuation [89–96]. Furthermore, modification of E83K in the serotype O FMDV VP1 protein blocks particle assembly [60] and results in an accretion of second site substitutions of L2P within the 2A protein, which also abrogates VP1/2A cleavage [61]. However, until now the effect of the E83K mutation in the VP1 protein on FMDV pathogenicity, and its impact on the cellular type-I IFN pathway, remain unclear.

In this study, we report a novel mechanism used by FMDV VP1 to avoid host immune responses and we examined the impact of the VP1 E83K substitution on FMDV pathogenicity. First, we showed that overexpression of FMDV VP1(83E) in epithelial cells reduced RNA virus-induced production of IFN-β and proinflammatory cytokines, and promoted viral replication, as shown previously [55,56]. However, this was not true for FMDV VP1(83K). Second, FMDV VP1(83E) interacted specifically with MAVS via aa 450–470, thereby inhibiting its interaction with TRAF3 via competitive binding; this inhibited IFN signaling and cellular antiviral responses. However, FMDV VP1(83K) lost binding to MAVS and retained MAVS interaction with TRAF3. Third, we generated chimeric FMDV harboring VP1 with an E83K substitution and evaluated its pathogenicity alongside that of FMDV harboring wild-type VP1 in suckling mouse and swine infection models. FMDV harboring VP1(83K) showed a significant reduction in pathogenicity but could still induce meaningful levels of neutralizing antibodies. Taken together, these findings indicate that FMDV VP1 inhibits host type-I IFN signaling, and that the E83K mutation within VP1 attenuates FMDV.

Previously, Li *et al*. reported that FMDV VP1 plays a role in suppression of type-I IFN responses by incorporation with sorcin [55]. They showed that FMDV VP1 inhibits TNF-α-induced or Sendi virus-induced type-I IFN responses and enhancement of VSV replication. They suggest the interaction with sorcin as a mechanism that explains FMDV VP1-induced IFN suppression [55]. However, the exact role of sorcin in the type-I IFN signaling pathway is still not clear. We also observed similar effects regarding FMDV VP1-induced IFN suppression, but our data suggest that this relates to a specific mechanism which target MAVS.

After confirming IFN inhibition phenotypes based on FMDV VP1 expression, we hypothesized that FMDV VP1 targets MAVS to inhibit type-I interferon signaling. FMDV VP1 did not affect IFN-β luciferase activity mediated by molecules downstream of MAVS (Fig 2G–2I). Also, interaction assays revealed that FMDV VP1 binds to MAVS (Fig 3B). However, FMDV VP1(83K) did not inhibit luciferase activity mediated by RIG-I or MAVS (Fig 2H–2J), and did not interact with MAVS (Fig 3C). MAVS acts as a critical adaptor protein of activated RLRs, which connect upstream viral RNA recognition to downstream signaling molecules to induce antiviral signaling [42,43,46,47]. MAVS comprises multiple domains: C-terminal transmembrane domain (TM), TRAF-interacting motifs, N-terminal proline-rich domains, and an N-terminal CARD domain [63]. Each domain plays a critical role in MAVS-mediated signaling; in particular, the TRAF-interacting motifs interact with downstream TRAF2, TRAF3, or TRAF6 molecules [62,63]. TRAF3 binds to aa 455–460 of MAVS to regulate downstream type-I IFN signaling [62,63]. Here, we found that FMDV VP1(83E) interacts specifically with the C-terminal TRAF3-binding site of MAVS (aa 455–460) [62,63], and that this interaction overlaps the VP1 binding site within MAVS (aa 450–470); thus VP1 and TRAF3 compete for binding to MAVS, leading to type-I IFN pathway suppression. This is the same mechanism reported previously for cellular UBXV1, which binds to aa 438–467 of MAVS and attenuates the MAVS/TRAF3 interaction [97]. However, this phenomenon was not observed in cell culture-adapted VP1 E83K.

Consequently, recombinant FMDV harboring VP1(83K) showed a significant reduction in pathogenicity in suckling mouse (Fig 4F) and swine models (Fig 5). Similarly, previous studies show that mutations in viral proteins after cell culture-adaptation disable their suppression ability of type-I IFN signaling, resulting attenuated viral pathogenicity. As an example, the A30P single amino acid substitution in the West Nile virus NS2A non-structural protein disables its ability to inhibit type-I IFN induction and attenuates virulence in mice [98]. Also, an E96A/E97A NS1 mutant of influenza A virus is defective in blocking TRIM25-mediated antiviral IFN responses, leading to lost virulence in mice [99]. Additionally, two point mutations (K319A/R322A) in the Ebola virus VP35 protein render the Ebola virus avirulent in guinea pigs because it cannot suppress IFN responses [100]. Based on these findings, we suggest that the low pathogenicity of the chimeric cell culture-adapted FMDV containing VP1(83K) is due to alteration of receptor specificity and a subsequent inability to suppress type-I IFN.

In addition, the chimeric FMDV containing VP1(83K) induced a higher neutralizing antibody titer, with no severe clinical signs. Nevertheless, FMDV harboring VP1(83K) and FMDV harboring VP1(83E) showed similar growth rates in cell culture system, even though VP1(83K) failed to suppress type-I IFN responses. We think that the selective advantage gained from the receptor alteration due to VP1 83K mutation in the cell culture system might be more dominant characteristic for viral replication and we assume that is the reason why pOm-AD-83E and pOm-AD-83K chimeric viruses show similar growth rates in cell culture system.

Importantly, since the FMDV VP1 83K point mutation which can be gained naturally from the cell culture adaptation results in virus attenuation, and the cell culture adaptation up to five passages results only VP1 83K point mutation in the structural protein (P1) region of FMDV; the cell culture model can be used for virus attenuation which could be utilized for the future development of FMDV vaccines.

In summary, we not only describe the immune evasion mechanism used by FMDV VP1, which is crucial for viral pathogenicity, but also show that the E83K mutation in the VP1 region attenuates the virus by altering its ability to recognize its cognate receptor and removing its ability to suppress the type-I IFN pathway. These observations may stimulate the search for additional mechanisms by which FMDV evades host IFN responses, and suggest a rational approach to virus attenuation during preparation of future FMD vaccines.

## Materials and methods

### Ethics statement

Animal experiments were performed in strict accordance with the recommendations of the guide for the care and use of laboratory animals of the Animal and Plant Quarantine Agency (APQA). All animal procedures were approved by the Institutional Animal Care and Use Committee of the APQA of Republic of Korea (approval no. 2015–02). All efforts were made to minimize animal suffering.

### Cells and antibodies

HEK293T, PK-15, Vero, BHK-21, LFBK, IBRS-2 cells were cultured in Dulbecco’s Modified Eagle’s Medium (DMEM-high glucose) (Gibco) containing 10% heat-inactivated fetal bovine serum (Gibco) and 1% antibiotic/antimycotic solution (Gibco). ZZ-R cells were sustained in DMEM F-12 (Corning) with 10% FBS. Cells were incubated at 37°C with 5% CO_2_ atmosphere. For western blot analysis, specific antibodies for Flag (M2) (8146) were purchased from Cell Signaling Technology and GST (sc-138) were purchased from Santa Cruz Biotechnology. Antibodies for Strep (2-1509-001) was purchased from IBA Life Sciences.

### Plasmids

Full form MAVS and its mutants carrying each domain expressing plasmids were cloned into a pEBG vector tagged with GST. To generate VP1 different construct, Wild-type and point mutated (E83K) VP1 was amplified from template DNA using PCR and cloned into pIRES-Flag, pEXPR-Strep vectors. 2CARD domain of RIG-I, RIG-I, TRAF3 plasmid constructs were obtained by amplification of template DNA using PCR and cloned into pIRES-Flag vector. The generation of the IFN-β promoter, luciferase reporter plasmids have been described elsewhere [101].

### Virus infection and plasmid transfection

GFP-expressing vesicular stomatitis virus (VSV-GFP) was propagated in the Vero cells and titrated by plaque assay. Before virus infection into the cells, the culture medium was changed with DMEM containing 1% FBS and 1% antibiotic-antimycotic, and infected into target cells with multiplicity of infection (MOI). After 2hr incubation at 37°C, extracellular virus was removed and replaced with 10% FBS containing DMEM. Plasmids were transfected to HEK293T and PK15 cells with Lipofectamine 2000 (Invitrogen) according to the manufacturer’s protocol.

### Virus titer determination

VSV-GFP infected cell culture supernatants were collected for the indicated times and virus titers were measured by plaque assay using *Ceropithecus aethiops* epithelial kidney (Vero) cells. Monolayer of Vero cells were seeded in 12-well plates and following 12hr of incubation, cells were inoculated for 2hr with serially diluted virus containing culture supernatants with 1% DMEM. After 2hr incubation, inoculums were removed and replaced with DMEM containing 0.1% agarose (Sigma-Aldrich). Plates were then incubated at 37°C for another 36hr and examined for plaque formation under 200x magnification. Virus titer was calculated using the number of plaque forming units and the dilution factor.

### ELISA

ELISA is performed to detect secreted flow inflammatory cytokines in cell culture supernatants. Human interferon-β (CUSABIO, CSB-E09889h) and porcine IFN-β (CUSABIO, CSB-E09890p) were used for analysis according to the manufacturer’s protocols.

### Quantitative real-time PCR

FMD virus wild-type and E83K substituted VP1 or pIRES empty vector overexpressed PK-15 cells were grown in 6-well tissue culture plates (1 × 10^6^ cells/well) and incubated at 37°C. The cells were infected with VSV-GFP (MOI = 0.1) and the cells were harvested at 24 hpt. The total RNA from the cells was isolated using the RNeasy Mini Kit (Qiagen), and cDNA was synthesized using reverse transcriptase (Toyobo). The different levels of cDNA were quantified by real-time polymerase chain reaction (PCR) using a QuantiTect SYBR Green PCR kit (Toyobo) according to the manufacturer’s instructions on a Rotorgene (Qiagen). The primer sequences were as follow: pIFN-β, 5’- AAA TCG CTC TCC TGA TGT GT-3’(forward) and 5’- TGC TCC TTT GTT GGT ATC G-3’(reverse); pIFN-α, 5’- GCC TCC TGC ACC AGT TCT ACA-3’(forward) and 5’- TGC ATG ACA CAG GCT TC CA-3’(reverse); pIL-6, 5’- CAC CGG TCT TGT GGA GTT TC-3’(forward) and 5’- GTG GTG GCT TTG TCT GGA TT-3’(reverse); pISG-15, 5’- AAA TCG CTC TCC TGA TGT GT- 3’(forward) and 5’- TGC TCC TTT GTT GGT ATC G-3’(reverse); pMCP-1, 5’- CAG AAG AGT CAC CAG CAG CA-3’(forward) and 5’- TCC AGG TGG CTT ATG GAG TC-3’(reverse); pIL-1β, 5’- GGG ACT TGA AGA GAG AAG TGG-3’(forward) and 5’- CTT TCC CTT GAT CCC TAA GGT-3’(reverse); pPKR, 5’- GAG AAG GTA GAG CGT GAA G-3’(forward) and 5’- CCA GCA ACC GTA GTA GAG-3’(reverse); pMX-1, 5’- TAG GCA ATC AGC CAT ACG-3’(forward) and 5’- GTT GAT GGT CTC CTG CTT AC-3’(reverse) (Bioneer, Daejeon, Republic of Korea).

### GST pulldown and immunoprecipitation

Cells were harvested at 36 h post-transfection of target plasmids, and whole-cell lysates (WCL) were obtained after lysis with protease inhibitor cocktail and phosphatase inhibitor cocktail (Sigma) containing radio-immunoprecipitation assay (RIPA) lysis buffer (50mM Tris-HCl, 150mM NaCl, 0.5% sodium deoxycholate, 1% IGEPAL, 1mM NaF, 1mM Na_3_VO_4_) and sonication with a sonicator (Sonics). The WCL were precleared with Sepharose 6B (GE Healthcare Life Science) at 4°C for 2h. After pre-clearing, for GST pulldown, the WCL were incubated with a 50% slurry of glutathione-conjugated Sepharose (GST) beads (Amersham Biosciences) for 12h. The immunoprecipitated beads collected after centrifugation were washed with lysis buffer under different washing conditions.

### Immunoblot analysis

Harvested cells were lysed with radio-immunoprecipitation assay (RIPA) lysis buffer. Cell lysates or samples prepared with immunoprecipitated beads were separated by SDS-PAGE and transferred on to a PVDF membrane using semi dry transfer cell (Bio-Rad, Seoul, Korea). Then, the membrane was blocked for 1 hour in 5% bovine-serum albumin and incubated overnight at 4°C with the primary antibody. Next day, membranes were washed with TBST or PBST and membrane was incubated with horseradish peroxidase-conjugated (HRP) secondary antibody for 2 hours at ambient temperature. Again, membrane was washed 3 times with TBST or PBST and finally, the reaction was visualized using an enhanced chemiluminescence detection system (ECL-GE Healthcare, Little Chalfont, United Kingdom) using a Las-3000 mini Lumino Image Analyzer.

### Luciferase reporter assay

HEK293T cells were cultured in 12 well tissue culture plates (3.5 × 10^5^ cells/well) and incubated at 37°C with 5% CO_2_ atmosphere, overnight. The cells in each well were transfected with 400ng luciferase reporter plasmid (IFN-β) and 10ng of TK-Renilla (an internal control for the normalization of the transfection efficiency) luciferase reporter plasmid together with Flag-tagged FMDV VP1 plasmid dose-dependently or control vector. The plasmids encoding RIG-I, 2CARD, MAVS, TRAF3, TBK1, and IKK-ε was cotransfected to stimulate the cells. Plasmids were transfected by using PEI reagent. At 24h post-transfection, cells were washed with PBS and lysed with 1X Passive Lysis buffer (Promega) for 20 minutes. Luciferase activity was measured using Dual-Luciferase Reporter Assay System (Promega; E1980) following manufacturer’s instruction. Luciferase activity in cells expressing only IFN-β reporter and Renilla plasmids was measured as a control. Data are expressed in accordance with relative firefly luciferase activity normalized against Renilla luciferase activities.

### Rescue of chimeric viruses

The pOm-83K is the cell culture adapted O1/Manisa/Turkey/69 (O1 Manisa) FMDV strain which has resulted in VP1 83K point mutation naturally throughout the cell culture adaptation. The pOm-83E was produced from mutagenesis by changing the Lysine (K) to Glutamic acid (E) at the 83rd amino acid of the VP1 of pOm-83K by using the KOD-Plus-Mutagenesis Kit (TOYOBO), and the following primers; 5’-CAC GAG GGA AAC CTC ACC TG- 3’ (forward) and 5’–CTT CAC TGC CAC CTC TAA GT-3’ (reverse). For the pOm-AD-83E virus generation, the P1 region of O/Andong/SKR/2010 (Andong) FMDV strain which present VP1 83E was secured by PCR using Phusion high-fidelity DNA polymerase (Thermo Fisher Scientific) according to the manufacturer’s instructions. Then the O1 Manisa backbone was PCR amplified as an insertion vector, except for their P1 region from the full-length infectious cDNA clones of O1 Manisa which was constructed previously [102]. The resultant O1 Manisa backbone was ligated with a secured P1 region of Andong strain by using a TaKaRa Long DNA ligation kit. For the generation of pOm-AD-83K, we performed mutagenesis for the mutation from E to K in the pOm-AD-83E construct by using the following primers; 5’ -CAC AAG GGG GAC CTT ACC TG -3’ (forward) and 5’ -TTT CAC TGC CAC CTC TAA ATC-3’ (reverse). The cloned plasmids were linearized by treatment with the restriction enzyme SpeI (NEB). Then the BHK T7-9 (baby hamster kidney) cells that stably express T7 RNA polymerase were transfected by using Lipofectamine 2000 (Invitrogen) with these linearized plasmids to recover chimeric viruses. These chimeric viruses were then amplified in the ZZ-R fetal goat tongue cell line for the isolation of recovered chimeric viruses [103]. The mutation was confirmed through the full genome sequencing of plasmid clones and recovered viruses.

### One-step growth and plaque assay in various cells

One-step growth curves and plaque assays for the pOm-AD-83K virus and pOm-AD-83E virus were conducted in BHK-21, LFBK, and IBRS-2 cells. To generate one-step growth curves, the cells were inoculated in a 12-well plate and incubated overnight, followed by inoculation of each virus at 0.1 multiplicity of infection (MOI). Supernatants of each culture were collected at 0, 2, 4, 8, 12, and 24 hr, and viral RNAs were extracted from the collected supernatants, followed by real-time RT-PCR using specific primers.

For plaque assays, the cells were inoculated in a 6-well plate and incubated overnight, followed by inoculation of each virus in each well at 10^5.0^~10^1.0^ TCID_50_/0.1mL with 10-fold dilutions. Each well was stained with crystal violet at 72 hr after virus inoculation, and the size of the plaque was determined.

### Pathogenesis in pigs

Viremia and clinical scores obtained from sera or swabs after the challenge of pOm-AD-83K virus and pOm-AD-83E in pigs. The challenge experiment was carried out with six 90-day-old Yucatan pigs. Three pigs were inoculated with pOm-AD-83K and the other three pigs were inoculated with pOm-AD-83E. Each virus was inoculated on the footpad at a concentration of 10^5.0^ TCID_50_/0.1mL. Sera and swab samples were collected daily from the six pigs from 0 days post-challenge (dpc) to 10 dpc, and body temperature and clinical symptoms were also monitored. For viremia analysis, viral RNAs were extracted from the sera and swab samples and then real-time RT-PCR was performed using specific primers. The clinical score was determined as follows: an elevated body temperature of 40°C (1 point), >40.5°C (2 points), or >41°C (3 points); reduced appetite (1 point) or no food intake and food leftover from the day before (2 points); lameness (1 point) or reluctance to stand (2 points); the presence of heat and pain after palpation of the coronary band (1 point) or not standing on the affected foot (2 points); vesicles on the feet, dependent on the number of feet affected, with a maximum of 4 points; and visible mouth lesions on the tongue (1 point), gums or lips (1 point), or snout (1 point), with a maximum of 3 points [104].

### Virus neutralization test

Serum samples were collected from pigs after inoculated and were heat-inactivated at 56°C for 30 min, Following the incubation of the test serum with FMDV 100TCID_50_ for 1 h, LFBK cells were added to the plate and incubated for 3 days. The CPE was checked to determine the titers, which were calculated as the log_10_ of the reciprocal antibody dilution to neutralize 100 TCID_50_ of the virus. FMDV O/Andong/SKR/2010 were used for VNT.

### Graphing and statistical analysis

Graphs and all Statistical analysis were performed using GraphPad Prism software version 6 for Windows. Data are presented as the means ± standard deviations (S.D.) and are representative of at least three independent experiments. Unpaired t-test was performed at each time point to compare the control and treatment groups. *P < 0.05 or **P < 0.01 was regarded as significant.

## Supporting information

S1 Fig Wild-type FMDV VP1 inhibits poly(I:C) and 5’ppp-dsRNA stimulated IFN-β production. (A) PK15 cells were transfected with wild-type VP1(83E) and VP1 83K plasmids of FMDV O/Andong/SKR/2010 (Andong) strain, along with a control vector for 24h. Then the cells were treated with Poly(I:C) (1μg/ml) for another 24h. At 12 and 24h time points after Poly(I:C) treatment, cell supernatant was analyzed for IFN-β secretion. (B) Similar to A, the same experiment was conducted with 5’ppp-dsRNA (1μg/ml) and checked the IFN-β secretion. Data are representative of two independent experiments, each with similar results. All the values are expressed as mean ± SD of two biological replicates. Student’s t test; *p < 0.05; **p < 0.01; ***p < 0.001; ns, not significant.
(TIF)

S2 Fig FMDV VP1 does not inhibit downstream IFN signaling. (A and B) HEK293T cells were transfected with wild-type VP1(83E) and VP1 83K plasmids of FMDV O/Andong/SKR/2010 (Andong) strain, along with a control vector. At 24h post-transfection, cells were treated with 800U/ml IFN-β for 12h, and PR8-GFP virus (1MOI) was infected. At indicated times after virus infection (A) GFP expression, (B) GFP absorbance and virus titer were measured. Data are representative of two independent experiments, each with similar results. Error bars indicate the mean ± SD.
(TIF)

S3 Fig Wild-type FMDV VP1 interacts with MAVS in type-I IFN pathway. (A) HEK293T cells were cotransfected with the control vector (Flag), Flag-tagged RIG-I, MDA5, MAVS, TRAF3, and TBK1 plasmids together with Strep-tagged wild-type VP1(83E) plasmids of FMDV O/Andong/SKR/2010 (Andong) strain. Cell lysates were subjected to Flag pulldown (PD), followed by immunoblotting with an anti-Strep antibody. Whole-cell lysate (WCL) was immunoblotted with anti-Strep and anti-Flag antibodies. Data are representative of two independent experiments, each with similar results.
(TIF)

S4 Fig Wild-type FMDV VP1 interrupts MAVS and TRAF3 interaction. (A) HEK293T cells were transfected with Strep-tagged wild-type VP1(83E), VP1 83K plasmids of FMDV O/Andong/SKR/2010 (Andong) strain or control plasmid (Strep) together with Flag-tagged TRAF3 and V5-tagged MAVS plasmids, followed by confocal microscopy assay with anti-Flag (red) and anti-V5 (green) antibodies. Nuclei were stained with DAPI (blue). Images are representative of two independent experiments, each with similar results.
(TIF)

S5 Fig Recombinant virus growth in BHK-21, LFBK, and IBRS-2 cells. (A) BHK-21, LFBK, and IBRS-2 cells were infected with pOm-83K, pOm-83E, pOm-AD-83K, and pOm-AD-83E virus and virus titer were determined by RNA extraction and quantitative real-time PCR analysis. This process was followed for up to five passages in each cell.
(TIF)

S1 Table Comparison of VP1 amino acids of type O FMDV occurred in the world. The 83^rd^ amino acid is highlighted in red.
(XLSX)

S2 Table Cytopathic effects of rescued viruses in three different cells (BHK-21, LFBK and IBRS-2 cells). (XLSX)

S3 Table Amino acid change of the viruses after serial passage in BHK-21, LFBK and IBRS-2 cells. (XLSX)
