# Supplementary figures and images for: Foot-and-mouth disease virus VP1 target the MAVS to inhibit type-I interferon signaling and VP1 E83K mutation results in virus attenuation
Source: PLoS Pathog. 2020 Nov 24;16(11):e1009057. doi: 10.1371/journal.ppat.1009057 (PMC7723281; doi:10.1371/journal.ppat.1009057)

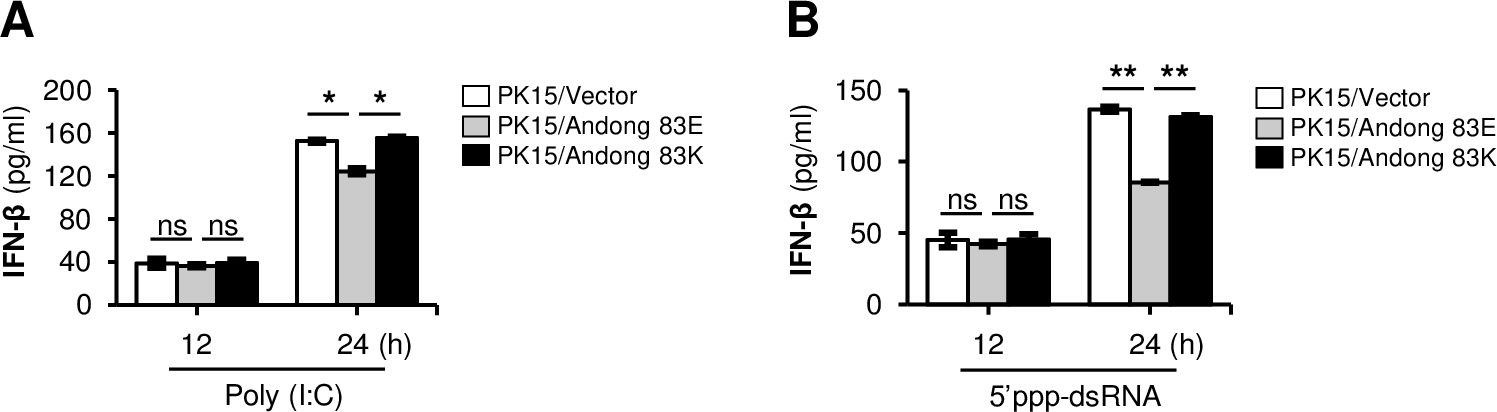

Supplement: S1 Fig — (A) PK15 cells were transfected with wild-type VP1(83E) and VP1 83K plasmids of FMDV O/Andong/SKR/2010 (Andong) strain, along with a control vector for 24h. Then the cells were treated with Poly(I:C) (1μg/ml) for another 24h. At 12 and 24h time points after Poly(I:C) treatment, cell supernatant was analyzed for IFN-β secretion. (B) Similar to A, the same experiment was conducted with 5’ppp-dsRNA (1μg/ml) and checked the IFN-β secretion. Data are representative of two independent experiments, each with similar results. All the values are expressed as mean ± SD of two biological replicates. Student’s t test; *p < 0.05; **p < 0.01; ***p < 0.001; ns, not significant. (TIF) [file ppat.1009057.s001.tif]

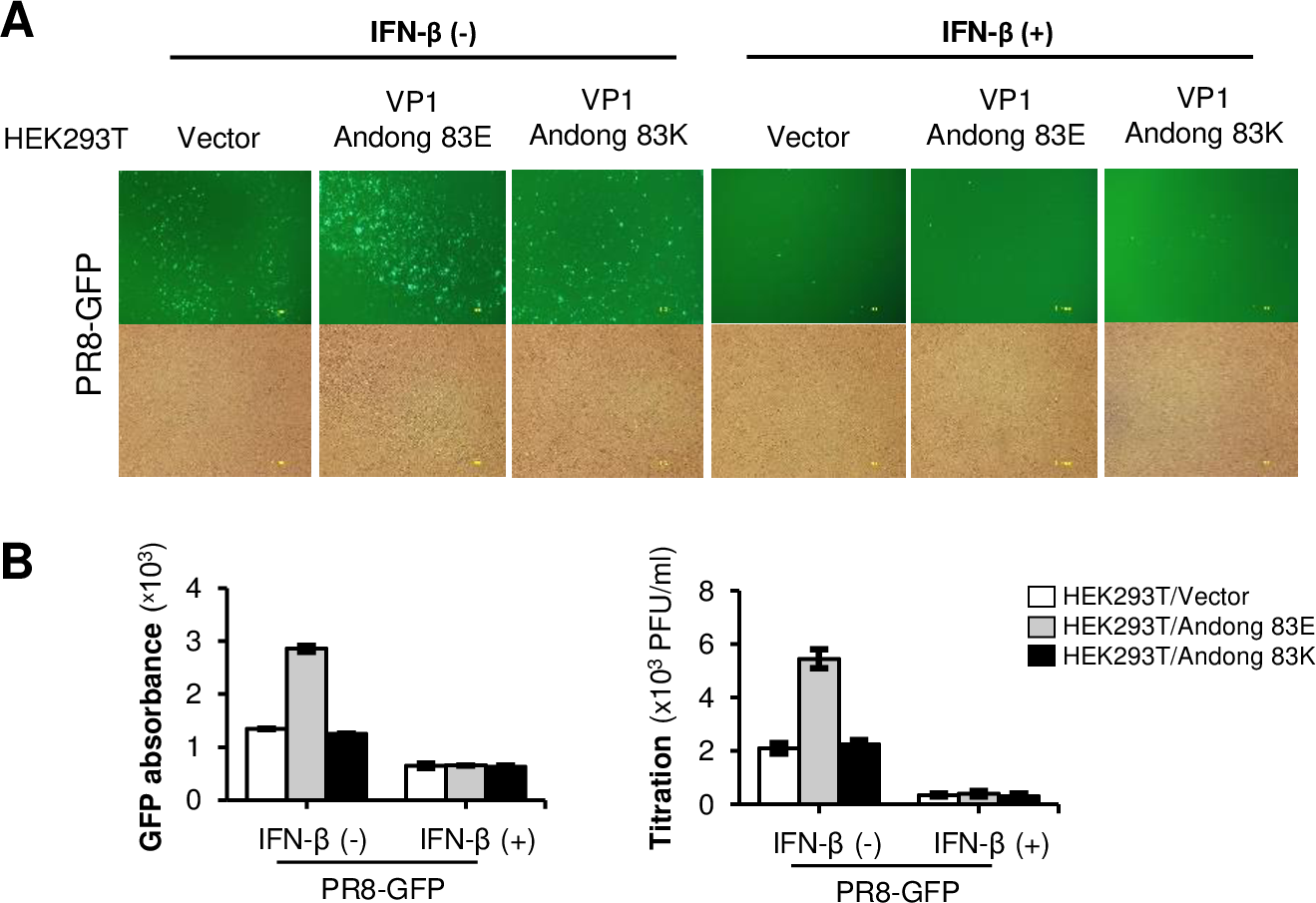

Supplement: S2 Fig — (A and B) HEK293T cells were transfected with wild-type VP1(83E) and VP1 83K plasmids of FMDV O/Andong/SKR/2010 (Andong) strain, along with a control vector. At 24h post-transfection, cells were treated with 800U/ml IFN-β for 12h, and PR8-GFP virus (1MOI) was infected. At indicated times after virus infection (A) GFP expression, (B) GFP absorbance and virus titer were measured. Data are representative of two independent experiments, each with similar results. Error bars indicate the mean ± SD. (TIF) [file ppat.1009057.s002.tif]

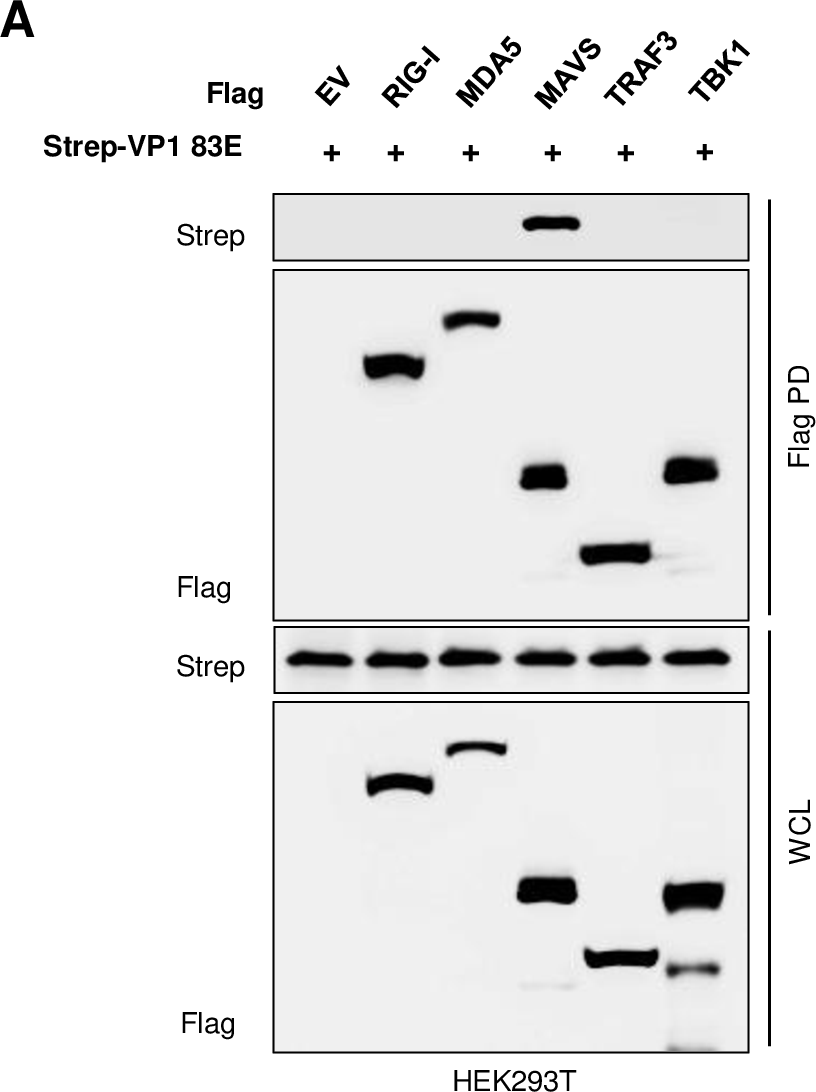

Supplement: S3 Fig — (A) HEK293T cells were cotransfected with the control vector (Flag), Flag-tagged RIG-I, MDA5, MAVS, TRAF3, and TBK1 plasmids together with Strep-tagged wild-type VP1(83E) plasmids of FMDV O/Andong/SKR/2010 (Andong) strain. Cell lysates were subjected to Flag pulldown (PD), followed by immunoblotting with an anti-Strep antibody. Whole-cell lysate (WCL) was immunoblotted with anti-Strep and anti-Flag antibodies. Data are representative of two independent experiments, each with similar results. (TIF) [file ppat.1009057.s003.tif]

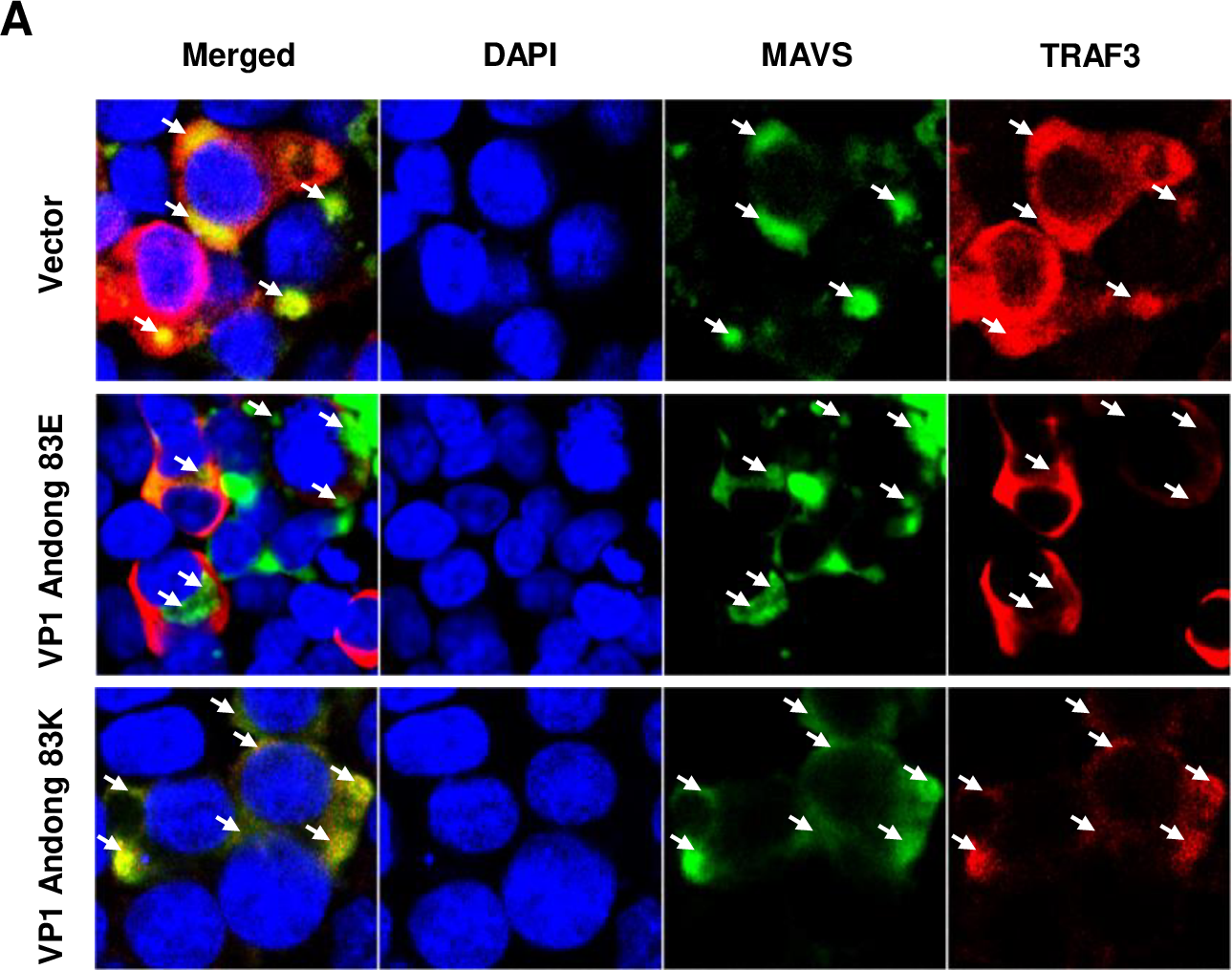

Supplement: S4 Fig — (A) HEK293T cells were transfected with Strep-tagged wild-type VP1(83E), VP1 83K plasmids of FMDV O/Andong/SKR/2010 (Andong) strain or control plasmid (Strep) together with Flag-tagged TRAF3 and V5-tagged MAVS plasmids, followed by confocal microscopy assay with anti-Flag (red) and anti-V5 (green) antibodies. Nuclei were stained with DAPI (blue). Images are representative of two independent experiments, each with similar results. (TIF) [file ppat.1009057.s004.tif]

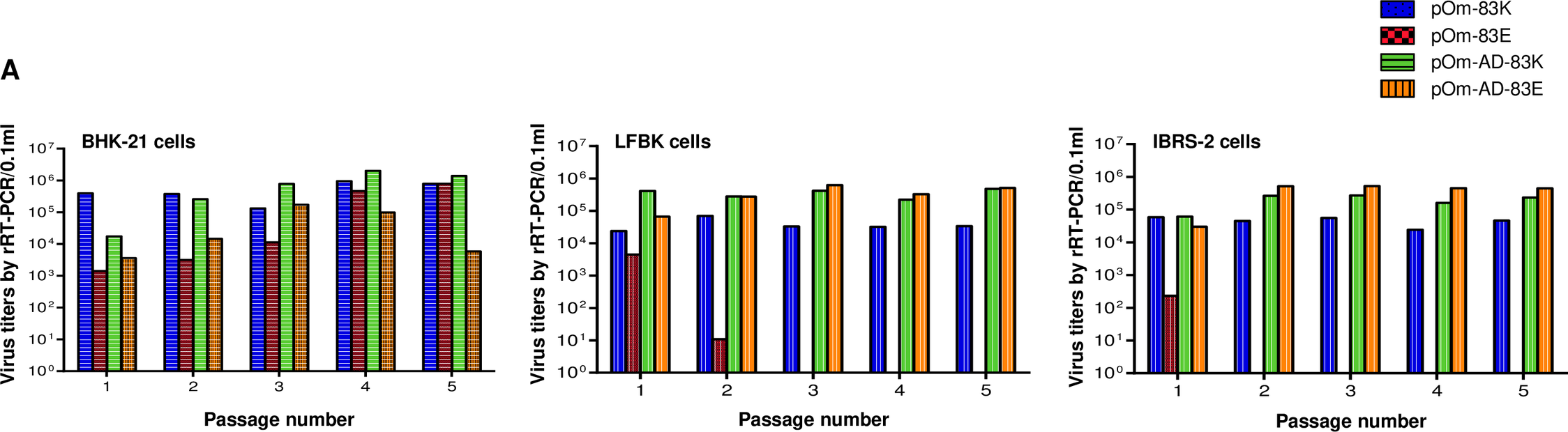

Supplement: S5 Fig — (A) BHK-21, LFBK, and IBRS-2 cells were infected with pOm-83K, pOm-83E, pOm-AD-83K, and pOm-AD-83E virus and virus titer were determined by RNA extraction and quantitative real-time PCR analysis. This process was followed for up to five passages in each cell. (TIF) [file ppat.1009057.s005.tif]
